# Enormous Empyema

**Published:** 1914-01

**Authors:** 


Enormous Empyema. Dr. D. H. Galloway, Roswell, N. Mex., *Am. Med.*, Oct. 1913. The bulging of the left chest could be seen through the clothes and, on direct inspection, three saucer-shaped tumors were noted, each six inches in diameter and two inches thick in the middle. One hundred and eighty-eight ounces were aspirated in the evening, to relieve dyspnoea and, the next day, a rib was resected and over a gallon of foul pus evacuated. The lung was seen as a lump, the size of a fist, near the apex. It never expanded and the heart never returned to its normal location but the patient survived nine months.

